# Loss of growth inhibitory effects of retinoic acid in human breast cancer cells following long-term exposure to retinoic acid

**DOI:** 10.1054/bjoc.2000.1388

**Published:** 2000-11

**Authors:** R Stephen, P D Darbre

**Affiliations:** Division of Cell and Molecular Biology, School of Animal and Microbial Sciences, The University of Reading, Whiteknights, P.O. Box 228, Reading, RG6 6AJ, England

**Keywords:** retinoic acid, breast cancer cells

## Abstract

Although retinoids are known to be inhibitory to breast cancer cell growth, a key remaining question is whether they would remain effective if administered long-term. We describe here the long-term effects of all-*trans* retinoic acid on two oestrogen-dependent human breast cancer cell lines MCF7 and ZR-75-1. Although both cell lines were growth inhibited by retinoic acid in the short-term in either the absence or the presence of oestradiol, prolonged culture with 1 μM all-*trans* retinoic acid resulted in the cells acquiring resistance to the growth inhibitory effects of retinoic acid. Time courses showed that oestrogen deprivation of the cell lines resulted in upregulation of the basal non-oestrogen stimulated growth rate such that cells learned to grow at the same rate without as with oestradiol, but the cells remained growth inhibited by retinoic acid throughout. Addition of 1 μM all-*trans* retinoic acid to steroid deprivation conditions resulted in reproducible loss of growth response to both retinoic acid and oestradiol, although the time courses were separable in that loss of growth response to retinoic acid preceded that of oestradiol. Loss of growth response to retinoic acid did not involve loss of receptors, ER as measured by steroid binding assay or RARα as measured by Northern blotting. Function of the receptors was retained in terms of the ability of both oestradiol and retinoic acid to upregulate pS2 gene expression, but there was reduced ability to upregulate transiently transfected ERE- and RRE-linked reporter genes. Despite the accepted role of IGFBP3 in retinoic acid-mediated growth inhibition, progression to retinoic acid resistance occurred irrespective of level of IGFBP3, which remained high in the resistant MCF7 cells. Measurement of AP1 activity showed that the two cell lines had markedly different basal AP1 activities, but that progression to resistance was accompanied in both cases by a lost ability of retinoic acid to reduce AP1 activity. These results warn of potential resistance which could arise on long-term treatment with retinoic acid in a clinical situation and echo the problems of progression to endocrine resistance. It seems that whatever the constraints imposed on growth, these cells have a remarkable ability to escape from growth inhibition. However, the ability of retinoic acid to delay progression to oestrogen resistance is encouraging for endocrine therapy, and the concentration-dependence of retinoic acid resistance suggests that progression is not absolute but could be manipulated by dose. © 2000 Cancer Research Campaign


*British Journal of Cancer* (2000) **83**(9), 1183–1191
© 2000 Cancer Research Campaign
doi: 10.1054/ bjoc.2000.1388, available online at http://www.idealibrary.com on IDEAL®

### Loss of growth inhibitory effects of retinoic acid in human breast cancer cells following long-term exposure to retinoic acid

**R Stephen and PD Darbre**

Division of Cell and Molecular Biology, School of Animal and Microbial Sciences, The University of Reading, Whiteknights, P.O. Box 228, Reading, RG6 6AJ, England

**Summary** Although retinoids are known to be inhibitory to breast cancer cell growth, a key remaining question is whether they would remain effective if administered long-term. We describe here the long-term effects of all-*trans* retinoic acid on two oestrogen-dependent human breast cancer cell lines MCF7 and ZR-75-1. Although both cell lines were growth inhibited by retinoic acid in the short-term in either the absence or the presence of oestradiol, prolonged culture with 1 μM all-*trans* retinoic acid resulted in the cells acquiring resistance to the growth inhibitory effects of retinoic acid. Time courses showed that oestrogen deprivation of the cell lines resulted in upregulation of the basal non-oestrogen stimulated growth rate such that cells learned to grow at the same rate without as with oestradiol, but the cells remained growth inhibited by retinoic acid throughout. Addition of 1 μM all-*trans* retinoic acid to steroid deprivation conditions resulted in reproducible loss of growth response to both retinoic acid and oestradiol, although the time courses were separable in that loss of growth response to retinoic acid preceded that of oestradiol. Loss of growth response to retinoic acid did not involve loss of receptors, ER as measured by steroid binding assay or RARα as measured by Northern blotting. Function of the receptors was retained in terms of the ability of both oestradiol and retinoic acid to upregulate pS2 gene expression, but there was reduced ability to upregulate transiently transfected ERE- and RRE-linked reporter genes. Despite the accepted role of IGFBP3 in retinoic acid-mediated growth inhibition, progression to retinoic acid resistance occurred irrespective of level of IGFBP3, which remained high in the resistant MCF7 cells. Measurement of AP1 activity showed that the two cell lines had markedly different basal AP1 activities, but that progression to resistance was accompanied in both cases by a lost ability of retinoic acid to reduce AP1 activity. These results warn of potential resistance which could arise on long-term treatment with retinoic acid in a clinical situation and echo the problems of progression to endocrine resistance. It seems that whatever the constraints imposed on growth, these cells have a remarkable ability to escape from growth inhibition. However, the ability of retinoic acid to delay progression to oestrogen resistance is encouraging for endocrine therapy, and the concentration-dependence of retinoic acid resistance suggests that progression is not absolute but could be manipulated by dose. © 2000 Cancer Research Campaign

**Keywords:** retinoic acid; breast cancer cells

Retinoids have been reported to inhibit the growth of several breast cancer cell lines in culture (Lotan, 1979; Lacroix and Lippman, 1980; Marth et al, 1985; Wetherall and Taylor, 1986; Fontana et al, 1990) and to reduce breast tumour growth in animal models (Rettura et al, 1975; Moon et al, 1976; Grubbs et al, 1977; Moon et al, 1977). Furthermore, retinoic acid can augment the action of other breast cancer cell growth inhibitors both in vitro (Wetherall and Taylor, 1986; Fontana, 1987; Koga and Sutherland, 1991) and in vivo (Anzano et al, 1994). Following the success of all-*trans* retinoic acid in differentiation therapy for acute promyelocytic leukaemia (Kizaki et al, 1999; Slack 1999), interest has increased in the potential use of retinoids for the prevention (Costa, 1993) and treatment (Budd et al, 1998) of human breast cancer. However, most of the reports of cell culture experiments have exposed cells to retinoic acid for relatively short periods of time (days). The key remaining question is whether retinoids would still be effective against breast cancer cells if administered long-term. There are reports of retinoic acid resistance in some breast cancer cell sublines (Lacroix et al, 1984; Ueda et al, 1985; Butler and Fontana, 1992) but no indication as to whether these result from rare mutational events or as a general adaptive consequence of long term exposure to retinoic acid.

*Received 14 February 2000*
*Revised 6 June 2000*
*Accepted 8 June 2000*

*Correspondence to:* PD Darbre

Endocrine treatment is well known to be limited by the problem of progression to resistance (Miller, 1996). Studies of oestrogen dependent human breast cancer cell lines in vitro show that such cells have a remarkable ability to adapt to their environment such that whatever the constraints imposed on growth, the cells learn to escape growth inhibition and to regrow. This can occur whether growth inhibition is imposed by oestrogen deprivation (Katzenellenbogen et al, 1987; Welshons and Jordan, 1987; Daly and Darbre, 1990; Jeng et al, 1998) or antioestrogen administration (Wakeling, 1993; Larssen et al, 1997). Much has been talked of as oestrogen resistance but insofar as such cells retain oestrogen receptor (ER) and oestrogen sensitive gene expression, the cells only develop proliferative resistance to oestrogen.

Since overall growth of breast cancer cells results from a delicate balance of cross-talk between different growth regulatory pathways including ligands of the nuclear receptor superfamily and various growth factors, resistance to one pathway has been suggested to result from upregulation of alternative pathways. There are several reports that retinoic acid action may be dependent on other oestrogen and growth factor pathways. Retinoic acid acts by binding to its nuclear receptors, the RARα, RARβ, RARγ, and as the 9-cis isomer through the retinoid X receptors, RXRα, RXRβ, RXRγ, to regulate gene expression at specific response elements (RRE) (Chambon, 1996). Expression of RARα, RARβ and RARγ have been found in many human breast cancer cell lines, but RARα is higher in ER positive lines (Roman et al, 1992). In these cells, oestrogen can regulate expression of RARα mRNA (Roman et al, 1993), probably through the oestrogen response element (ERE) upstream of the RARα gene (Rishi et al, 1995). Experiments with selective retinoids (Dawson et al, 1995) and with transfecting ER/RARα genes into ER negative cell lines (Sheikh et al, 1993, 1994) suggest that RARα is central to the mechanism of retinoic acid-induced growth inhibition in breast cancer cells, although the importance of other retinoid receptors cannot be ignored (Li et al, 1995). While oestrogen can regulate RARα mRNA, conversely retinoic acid can downregulate ER mRNA (Rubin et al, 1994) and can also inhibit downstream oestrogen-induced gene expression (Fontana et al, 1992; Kazmi et al, 1996), suggesting a two-way interactive regulatory pathway. On the growth factor side, retinoic acid has been found to inhibit proliferation mediated through insulin-like growth factors (IGF) (Fontana et al, 1991), by increasing the secretion of IGF binding proteins (IGFBP) and in particular of IGFBP3 (Adamo et al, 1992; Oh, 1998) through a mechanism dependent on both RARα and RARβ (Shang et al, 1999).

This manuscript provides evidence that endocrine resistance is not a unique phenomenon and that resistance to retinoic acid-induced growth inhibition can also occur in vitro when cells are exposed long term to this compound. Resistance occurs reproducibly, without the loss of ER or RARα, and irrespective of the level of IGFBP3.

### MATERIALS AND METHODS

### Cell lines

MCF7 McGrath human breast cancer cells were kindly provided by Dr K Osborne at passage number 390 (Osborne et al, 1987) and ZR-75-1 human breast cancer cells were kindly provided by Professor M. Lippman (Engel et al, 1978). Both cell lines are dependent on oestrogen for growth as described previously (Darbre and Daly, 1989).

### Culture of stock human breast cancer cell lines

Stock MCF7 cells were grown as monolayer cultures in Dulbecco's modified Eagle's medium (DMEM) (Gibco BRL) supplemented with 5% fetal calf serum (FCS) (Gibco BRL), 10 μg/ml insulin (Sigma, Poole, England) and $10^{-8}$ M oestradiol (Steraloids, Croydon, England) in a humidified atmosphere of 10% carbon dioxide in air at 37°C. Oestradiol was dissolved in ethanol and diluted 1/10 000 (v/v) in culture medium. ZR-75-1 cells were grown routinely as for MCF7 cells except for the omission of insulin from the medium. All cell stocks were subcultured at weekly intervals by suspension with 0.06% trypsin-0.02% EDTA (pH 7.3).

### Culture of long-term oestrogen-deprived and retinoic acid-treated cells

A new vial of cells was thawed from liquid nitrogen at the start of each experiment which ensured that control cells of the starting passage number were available for comparison at any time. Freshly thawed cells were grown for 2 weeks as stock cultures with oestrogen (see above) and then suspended with phenol-red-free 0.06% trypsin-0.02% EDTA (pH 7.3), washed with phenol-red-free RPMI 1640 medium (Gibco BRL) and replated in phenol-red-free RPMI 1640 medium containing 5% dextran-charcoal stripped FCS (DCFCS) (Darbre et al, 1983) with either no further additions (for oestrogen-deprived conditions) or $10^{-6}$ M all-*trans*-retinoic acid (retinoic acid treated cells). All-*trans*-retinoic acid was dissolved at $10^{-2}$ M in dimethylsulphoxide and diluted 1/10 000 (v/v) in culture medium. All experiments with all-*trans*-retinoic acid were conducted under minimal light conditions. For the study of whole cell populations, cells were subcultured using phenol red-free 0.06% trypsin-0.02% EDTA (pH 7.3) as necessary after 1 week, thereafter every 2–3 weeks during the period of low growth and then increasing until eventually weekly. For the study of clonal growth of cells, medium was changed every 3–4 days but the cells were never subcultured. Well-separated clones of cells were isolated by trypsinization using cloning rings. Samples of whole cell cultures and individual clones were frozen in liquid nitrogen at regular intervals for comparison.

### Cell growth experiments

Cells were suspended from stock plates by treatment with phenol red-free 0.06% trypsin-0.02% EDTA (pH 7.3), added to an equal volume of phenol red-free RPMI 1640 medium containing 5% DCFCS and counted on a haemocytometer. Cells were then added to the required volume of phenol red-free RPMI 1640 medium containing 5% DCFCS at a concentration of $0.2 \times 10^5$ cells/ml and plated in monolayer in 0.5 ml aliquots into 24-well plastic tissue culture dishes (Nunc). After 24 hours, the medium was changed to phenol red-free RPMI 1640 medium supplemented with 5% DCFCS and the appropriate concentration of oestradiol and all-*trans*-retinoic acid. Culture medium was changed routinely every 3–4 days in all experiments. Cell counts were performed by counting released nuclei on a model ZBI Coulter Counter, as described previously (Daly and Darbre, 1990).

### RNA extraction and Northern blotting

Cells for RNA extraction were suspended in phenol red-free RPMI 1640 medium containing 5% DCFCS at a concentration of $0.2 \times 10^5$ cells/ml and plated in 16 ml aliquots into 9 cm plastic tissue culture dishes (Nunc). After 24 hours, the medium was changed to phenol red-free RPMI 1640 medium supplemented with 5% DCFCS and the appropriate concentration of oestradiol and all-*trans*-retinoic acid. After 7 days in culture, cells were washed in situ with isotonic saline, harvested into ice-cold isotonic saline using a rubber policeman and pelleted by centrifugation. Whole cell RNA was prepared by the guanidinium-caesium chloride method (Sambrook et al, 1989) and analysed by Northern blotting. Total cellular RNA was subjected to electrophoresis in 1.5% agarose-formaldehyde gels (Sambrook et al, 1989) at 20 μg RNA per track. RNA was transferred onto Hybond-N membranes (Amersham International) and hybridized to $10^6$ cpm of $^{32}P$

 © 2000 Cancer Research Campaign labelled DNA probe per ml. The RARα cDNA probe was a 1.8 kb Kpn1 fragment extending from the ATG to the BamH1 site in the 3′ non-coding region (kindly provided by R Evans) (Umesono et al, 1991). The pS2 cDNA probe was a 300-bp PstI fragment (Masiakowski et al, 1982) and the 36B4 control DNA probe was a 220-bp Pst1 fragment (Brown et al, 1984) (kindly provided by P Chambon). DNA probes were $^{32}$P-radiolabelled by random primer extension using a commercial kit (megaprime, Amersham International). Hybridization was at 42°C for 18 h in 5× SSPE/5× Denhardt's solution/50% formamide/0.5% SDS/20 μg/ml salmon sperm DNA. Blots were washed at a stringency of 0.1×SSPE/0.1% SDS at 65°C for 30 min and autoradiographed on Kodak XAR film with intensifying screens at –70°C (Daly and Darbre, 1990).

### Transient transfection assays

Three inducible constructs were used: RRE-LUC, ERE-CAT (Daly et al, 1990) (kindly provided by M Parker) and AP1-CAT (Soprano et al, 1996) (kindly provided by D Soprano). All constructs consisted of the relevant response element sequence inserted upstream of the thymidine kinase promoter and the reporter gene. Reporter genes were either chloramphenicol acetyl transferase (CAT) or firefly luciferase (LUC). Normalization of data was achieved by cotransfection with a control constitutive reporter construct. A constitutive CAT reporter gene was used to normalize RRE-LUC experiments, and a constitutive LUC reporter gene was used to normalize ERE-CAT and AP1-CAT experiments.

Cells were grown in monolayer culture in 3.5 cm tissue culture dishes in phenol red-free DMEM with 5% DCFCS from a density of $2.0 \times 10^5$ cells per dish for 7 days. Cells were then transfected for 6 h with 5 μg of inducible construct and 0.5 μg of control constitutive vector per dish using the calcium phosphate precipitation method (Wigler et al, 1979). Cells were washed in phenol red-free DMEM, shocked with 25% glycerol in DMEM for 1 min and incubated overnight in phenol red-free RPMI1640 medium with 5% DCFCS. The following day, the medium was changed to 5% DCFCS in phenol red-free RPMI1640 containing the required concentration of oestradiol and/or all-*trans*-retinoic acid. Cells were harvested 48 h later and assays for LUC activity performed using a commercial kit (Promega) and assays for CAT activity performed as described previously (Sleigh, 1986). All assays were performed in duplicate on triplicate dishes of cells.

### Western ligand blotting of IGFBP

Cells were plated onto 3.5-cm plastic tissue culture dishes in phenol red-free RPMI 1640 medium with 5% DCFCS and left to adhere overnight. After 24 hours the medium was changed to include $10^{-8}$ M oestradiol and/or $10^{-6}$ M all-*trans* retinoic acid as required. After 6 days, medium was changed to serum-free medium: cells were washed twice with phenol red-free RPMI1640 medium and incubated in 1 ml of serum-free medium per dish (phenol red-free RPMI 1640 medium with 15 mM HEPES buffer, 0.25% bovine serum albumin and any supplements of $10^{-8}$ M oestradiol and $10^{-6}$ M all-*trans* retinoic acid as above) for a further 24 hours. Medium conditioned by the cells was collected, cellular material removed by centrifugation, and medium stored at –70°C. Cells remaining on each dish were counted on a Coulter counter as above.

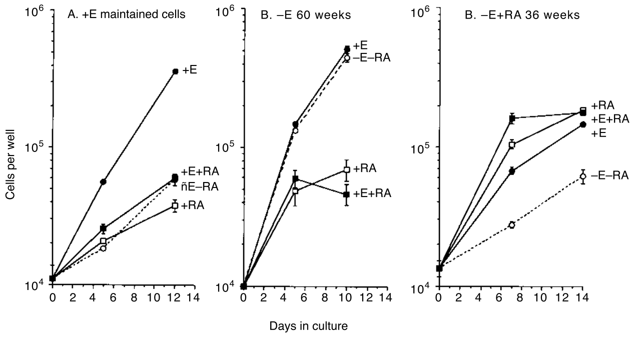

**Figure 1** Effect of long-term retinoic acid treatment on the growth regulation of MCF7 human breast cancer cells by oestradiol and all-*trans* retinoic acid in monolayer culture. Cells were maintained either as stock cultures with $10^{-8}$ M oestradiol (**A**), or under conditions of steroid deprivation (phenol red-free RPMI 1640/5% DCFCS only) (**B**), or under conditions of steroid deprivation with $10^{-6}$ M all-*trans* retinoic acid (**C**) for the length of time indicated. Short-term growth was then assessed in phenol red-free RPMI 1640/5% DCFCS medium alone (–E–RA) (open circles, dotted lines), or supplemented with $10^{-8}$ M oestradiol (+E) (solid circles, solid lines), with $10^{-6}$ M all-*trans* retinoic acid (+RA) (open squares, solid lines), with $10^{-8}$ M oestradiol and $10^{-6}$ M all-*trans* retinoic acid (+E+RA) (solid squares, solid lines). Bars indicate the standard error of triplicate dishes, and where not seen, error was too small for visual display

Aliquots of the conditioned medium were run on polyacrylamide gel electrophoresis, loading into each well the volume of conditioned medium equivalent to $10^5$ cells. Aliquots of conditioned medium were each mixed with an equal volume of gel sample buffer (26 mM Tris-HCl (pH 6.8), 2% sodium dodecyl sulphate (SDS), 10% glycerol, 0.01% bromophenol blue), heated to 100°C for 2 min and proteins separated by 15% polyacrylamide-SDS-gel electrophoresis. Proteins were transferred onto Hybond C extra membrane by semi-dry blotting in 48 mM Tris/ 39 mM glycine/1.3 mM SDS/20% methanol. Western blots were hybridized to $^{125}$I-IGFI as described by Hossenlopp and coworkers (1986). $^{125}$I-IGFI was prepared by the iodogen method (Salacinski et al, 1981).

### Measurement of oestrogen receptors (ER)

Freeze-fractured cell pellets from one 9-cm tissue culture dish were homogenized in the ratio 1:8 parts buffer (10 mM Tris-HCl, 1 mM EDTA, 2 mM DTT, 10% glycerol, 0.5 M NaCl, 4 mM leupeptin, pH 7.4) at 4°C with ten passes through a teflon-glass homogenizer. Homogenates were centrifuged at 105 000 ***g*** for 1 hour at 4°C in a Sorvall ultracentrifruge and cytosols stored at –70°C. Competitive binding assays using dextran-coated charcoal (DCC) were performed on the cytosols as described elswhere (Green and Leake, 1987). Incubations were performed for 18 hours at 4°C, and tubes were DCC-treated for 15 min at 4°C.

### RESULTS

### Short term effects of retinoic acid on growth of MCF7 cells

Oestrogen-maintained stock MCF7 cells grew slowly in the short-term absence of oestradiol but showed a strong stimulation of growth with addition of $10^{-8}$ M oestradiol. All-*trans* retinoic acid at $10^{-6}$ M inhibited both the basal and oestrogen-stimulated growth (Figure 1A). This growth experiment was repeated four times during the course of the experiment with similar results.

*© 2000 Cancer Research Campaign* 

### Long term effects of retinoic acid on growth of MCF7 cells

### *Experimental strategy*

Long term effects of retinoic acid were studied by growing stock cultures in oestrogen-depleted medium in the presence of $10^{-6}$ M all-*trans* retinoic acid. However, since oestrogen deprivation alone has been shown to have consequences to cell growth (Katzenellenbogen et al, 1987; Daly and Darbre, 1990), control stock cultures were maintained in parallel in oestrogen-depleted medium alone, in order to identify those effects specific to long term retinoic acid treatment. Cells were assayed from both stock cultures for cellular and molecular parameters at various time intervals.

### *Cell biology*

Long-term oestrogen deprivation of MCF7 cells resulted in a loss of oestrogen sensitive growth, such that the basal growth in the absence of oestradiol rose to the same rate as that in the presence of $10^{-8}$ M oestradiol. Growth of these oestrogen-deprived cells was, however, still strongly inhibited by the addition of $10^{-6}$ M all-*trans* retinoic acid, either in the absence or in the presence of $10^{-8}$ M oestradiol (Figure 1B). Long-term growth of the MCF7 cells for 36 weeks in the presence of $10^{-6}$ M all-*trans* retinoic acid under steroid deprived conditions preserved some oestrogen sensitivity of growth but showed a loss of growth inhibitory action by retinoic acid, in that all-*trans* retinoic acid at $10^{-6}$ M now stimulated growth of the cells either in the absence or in the presence of $10^{-8}$ M oestradiol (Figure 1C).

Time courses of the changes in the growth responses of the long-term steroid deprived cells and of the long-term retinoic acid-treated cells are shown in Figure 2. Steroid deprivation resulted in an increased growth rate in the absence of oestradiol such that by 15 weeks the cells grew to a similar extent irrespective of the absence or presence of $10^{-8}$ M oestradiol. Short-term growth response to $10^{-6}$ M all-*trans* retinoic acid increased during the course of the experiment but even after 98 weeks the steroid-deprived cells remained growth inhibited by retinoic acid (Figure 2A). Long-term growth of MCF7 cells under conditions of steroid deprivation has been carried out by two independent experiments with similar results.

Long-term growth in the presence of $10^{-6}$ M all-*trans* retinoic acid under steroid deprived conditions resulted in a loss of growth response to retinoic acid by 36 weeks. The cells remained growth stimulated by $10^{-8}$ M oestradiol but lost the inhibitory effect of $10^{-6}$ M all-*trans* retinoic acid at 36 weeks (Figure 1C, Figure 2B). Longer term growth of these latter cells, however, resulted eventually also in loss of growth response to $10^{-8}$ M oestradiol as well as to $10^{-6}$ M all-*trans* retinoic acid (Figure 2B). Study of the outgrowth of cells with long-term retinoic acid treatment showed, in unsubcultured dishes, that the adaptation to growth in the presence of retinoic acid was clonal but reproducibly gave several clones (4–6) per 9-cm culture dish. Studies of long-term retinoic acid treated cells were carried out on both subcultured stock dishes and on stock dishes never subcultured with clonal outgrowths individually isolated. Results in this paper are given for the clonal outgrowth of cells termed RR6, but similar time courses were obtained for subcultured dishes termed RRsub and a second clonal outgrowth of cells termed RR8 (data not shown here but described in a PhD thesis (Stephen, 1998)).

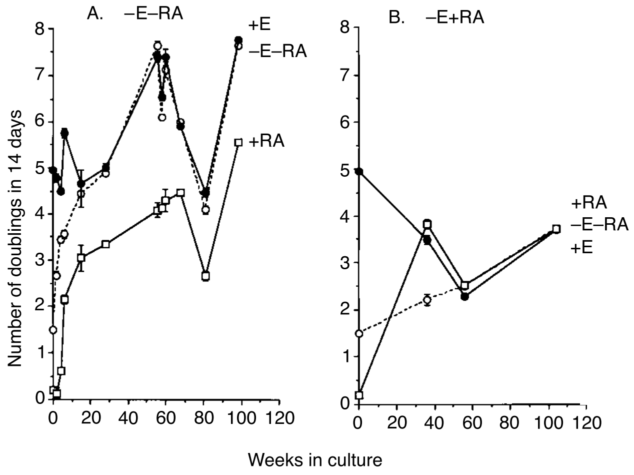

**Figure 2** Time course of the changes in growth response to oestradiol and all-*trans* retinoic acid of MCF7 human breast cancer cells in monolayer culture following long term retinoic acid treatment. Cells were grown for increasing periods of time under conditions of steroid deprivation (phenol red-free RPMI 1640/5% DCFCS only) (**A**), or under conditions of steroid deprivation with $10^{-6}$ M all-*trans* retinoic acid (**B**). Short-term growth was then assessed over 14 days in phenol red-free RPMI 1640/5% DCFCS medium alone (–E–RA) (open circles, dotted lines), or supplemented with $10^{-8}$ M oestradiol (+E) (solid circles, solid lines) or with $10^{-6}$ M all-*trans* retinoic acid (+RA) (open squares, solid lines). Bars indicate the standard error of triplicate dishes, and where not seen, error was too small for visual display

Loss of growth inhibition by $10^{-6}$ M all-*trans* retinoic acid in the long-term retinoic acid treated cells was accompanied by loss of inhibitory growth effects also of the 9-cis isomer. Long-term (103 weeks) retinoic acid treated cells were plated in a growth experiment at a density of $0.15 \times 10^5$ cells per dish and grew over 14 days to a density without retinoic acid of $2.001 \pm 0.001 \times 10^5$ cells per dish, with $10^{-6}$ M all-*trans* retinoic acid of $2.003 \pm 0.077 \times 10^5$ cells per dish, and with $10^{-6}$ M 9-cis retinoic acid of $2.027 \pm 0.046 \times 10^5$ cells per dish.

A separate experiment, however, suggested that loss of growth inhibition was a concentration-dependent phenomenon in that some growth inhibitory effects could still be detected by raising levels to $10^{-5}$ M all-*trans* retinoic acid. Long-term (56 weeks) retinoic acid treated cells were plated in a growth experiment at a density of $0.26 \times 10^5$ cells per dish and ended after 14 days at a density without retinoic acid of $1.885 \pm 0.0044 \times 10^5$ cells per dish, with $10^{-8}$ M all-*trans* retinoic acid of $1.928 \pm 0.155 \times 10^5$ cells per dish, with $10^{-7}$ M all-*trans* retinoic acid of $1.958 \pm 0.030 \times 10^5$ cells per dish, with $10^{-6}$ M all-*trans* retinoic acid of $1.981 \pm 0.036 \times 10^5$ cells per dish, but with $10^{-5}$ M all-*trans* retinoic acid of $1.369 \pm 0.070 \times 10^5$ cells per dish and with $10^{-4}$ M all-*trans* retinoic acid of $0.081 \pm 0.003 \times 10^5$ cells per dish.

### *Expression of ER and RARα*

Loss of growth response to oestradiol did not correlate with any loss of oestrogen receptor. Measurement of total salt-extractable ER in the stock oestrogen-maintained MCF7 cells gave a value of $7.5 \pm 1.5$ fmol/mg protein, as measured by competitive binding assay. Comparative assays showed increased levels of ER in MCF7 cells deprived of oestrogen for 110 weeks ($78.4 \pm 4.6$ fmol/mg protein) and in MCF7 cells treated with retinoic acid for 99 weeks ($44.4 \pm 1.1$ fmol/mg protein).

Since RARα has been implicated as the major mediator of proliferative effects of retinoic acid in ER positive breast cancer

 *© 2000 Cancer Research Campaign*

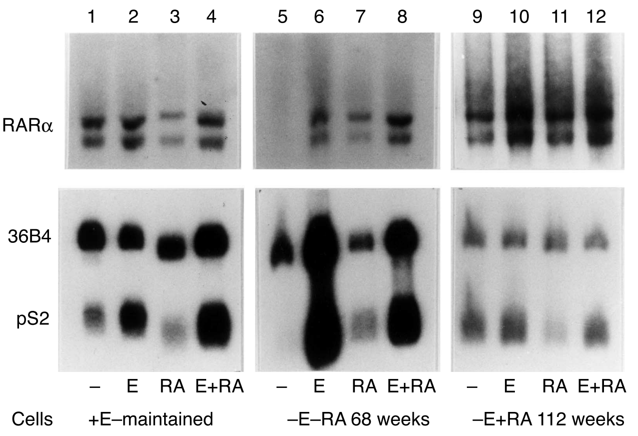

**Figure 3** Regulation of RARα mRNA in MCF7 human breast cancer cells following long-term retinoic acid treatment. Northern blots of whole cell RNA (20 µg per track) from stock oestrogen-maintained cells (tracks 1–4), or from cells following 68 weeks of steroid deprivation (phenol red-free RPMI 1640/5% DCFCS medium) (tracks 5–8), or from cells following 112 weeks of steroid deprivation in the presence of $10^{-6}$ M all-*trans* retinoic acid (tracks 9–12). Short-term regulation of gene expression was assessed in each stock line following growth for 7 days in RPMI 1640/5% DCFCS medium alone (–) (tracks 1, 5, 9), or supplemented with $10^{-8}$ M oestradiol (E) (tracks 2, 6, 10), with $10^{-6}$ M all-*trans* retinoic acid (RA) (tracks 3, 7, 11), or with $10^{-8}$ M oestradiol and $10^{-6}$ M all-*trans* retinoic acid (E+RA) (tracks 4, 8, 12). Blots were probed for RARα mRNA and oestrogen-regulated pS2 mRNA. Equal loading of RNA samples was controlled by probing for the constitutively expressed 36B4 mRNA

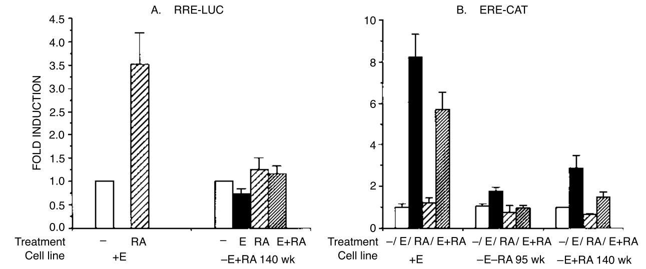

**Figure 4** Effect of long-term retinoic acid treatment on function of retinoic acid receptors (**A**) and oestrogen receptors (**B**) in MCF7 human breast cancer cells. Function of receptors was assayed by transient transfection of an RRE-LUC gene or of an ERE-CAT gene respectively. Cells were maintained either as stock cultures with $10^{-8}$ M oestradiol (cell line +E), or under conditions of steroid deprivation (phenol red-free RPMI 1640/5% DCFCS only) (cell line –E–RA), or under conditions of steroid deprivation with $10^{-6}$ M all-*trans* retinoic acid (cell line –E+RA) for the length of time indicated. Short-term expression of the transfected gene was then assessed as described in 'Materials and Methods' in phenol red-free RPMI 1640/5% DCFCS medium alone (–) (open bars), or supplemented with $10^{-8}$ M oestradiol (E) (solid bars), with $10^{-6}$ M all-*trans* retinoic acid (RA) (wide lines), with $10^{-8}$ M oestradiol and $10^{-6}$ M all-*trans* retinoic acid (E+RA) (close lines). Luciferase activity was expressed as optical density units of maximal light emission normalized per unit of CAT activity. CAT activity was expressed as pmols of $^{14}$C-acetyl group transferred from $^{14}$C-acetyl CoA to chloramphenicol per hour and normalized per unit of luciferase activity. All results are expressed as a fold induction ratio of treatment versus no treatment. Bars indicate the standard error of triplicate dishes of both no treatment and treatment cells (see introduction), we measured RARα mRNA levels by Northern blotting. Two mRNA species were found for RARα mRNA as documented by others (Roman et al, 1992). Levels of RARα mRNA did not vary to any large extent following short-term (7 day) removal of oestrogen or retinoic acid treatment of stock oestrogen-maintained MCF7 cells (Figure 3, tracks 1–4). However, longer term removal of oestrogen did result in loss of RARα mRNA (Figure 3, track 5) which could be reinduced with either $10^{-8}$ M oestradiol or $10^{-6}$ M all-*trans* retinoic acid (Figure 3, tracks 6–8). Long-term retinoic acid treatment resulted in retention of RARα mRNA at levels similar to those in the oestrogen-maintained cells and which again were not altered to any major extent by short term (7 day) removal of retinoic acid or oestrogen administration (Figure 3, tracks 9–12).

### *Function of oestrogen and retinoic acid receptors*

Although changes in growth responses were not accompanied by loss of ER or RARα, it is possible that downstream events in receptor function were altered. Transient transfection of an RRE-LUC gene into the oestrogen-maintained MCF7 cells confirmed that all-*trans* retinoic acid could induce luciferase activity in those cells, but following long-term retinoic acid treatment the induction of the same transfected gene was considerably reduced (Figure 4A). A similar result was obtained for induction of a transiently transfected oestrogen-inducible ERE-CAT gene (Figure 4B), in that the oestrogen induction of CAT activity was considerably reduced in long-term steroid deprived or retinoic acid treated cells compared with the oestrogen-maintained cells.

Other studies of oestrogen receptor function, by contrast, showed that oestrogen regulation of pS2 gene expression was not impaired in the long term steroid deprived and retinoic acid treated cells (Figure 3). Short-term removal of oestrogen (1 week) from oestrogen-maintained cells resulted in reduced pS2 mRNA levels which could be reinduced by oestradiol but not by retinoic acid (Figure 3, tracks 1–4). Longer term removal of oestrogen (68 weeks) resulted in undetectable levels of pS2 mRNA which could be reinduced by oestradiol and weakly also by retinoic acid (Figure 3, tracks 5–8). Long-term retinoic acid treatment preserved pS2 mRNA expression (Figure 3, cf tracks 5 and 9) although oestradiol could still increase levels further (Figure 3, tracks 9–12).

### Long term effects of retinoic acid on growth of ZR-75-1 cells

In order to determine general applicability of results from MCF7 cells, experiments were carried out in a second oestrogen-regulated human breast cancer cell line ZR-75-1. Stock oestrogen-maintained ZR-75-1 cells grew slowly in the short-term absence of oestrogen, and growth was stimulated by $10^{-8}$ M oestradiol and inhibited by $10^{-6}$ M all-*trans* retinoic acid (Figure 5A). Effects of long-term oestrogen deprivation in these cells have been described previously (Daly and Darbre, 1990). Long-term retinoic acid treatment of these cells under conditions of steroid deprivation resulted in loss of growth response to either oestradiol or all-*trans* retinoic acid (Figure 5B). Basal growth of the cells in the absence of oestrogen rose to the same rate as that in the presence of oestradiol and growth was no longer inhibited by $10^{-6}$ M all-*trans* retinoic acid. Previous work (Daly and Darbre, 1990) has shown that long term oestrogen deprivation does not result in loss of ER number or in loss of ER function as measured by oestrogen regulation of pS2 mRNA gene expression on Northern blotting. Long-term retinoic

© 2000 Cancer Research Campaign 

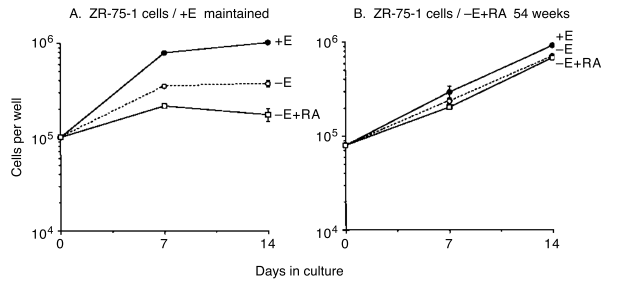

**Figure 5** Effect of long-term retinoic acid treatment on the growth regulation of ZR-75-1 human breast cancer cells by oestradiol and all-*trans* retinoic acid in monolayer culture. Cells were maintained either as stock cultures with $10^{-8}$ M oestradiol (**A**), or under conditions of steroid deprivation (phenol red-free RPMI 1640/5% DCFCS) with $10^{-6}$ M all-*trans* retinoic acid (**B**) for the length of time indicated. Short-term growth was then assessed in phenol red-free RPMI 1640/5% DCFCS medium alone (–E–RA) (open circles, dotted lines), or supplemented with $10^{-8}$ M oestradiol (+E) (solid circles, solid lines) or with $10^{-6}$ M all-*trans* retinoic acid (+RA) (open squares, solid lines). Bars indicate the standard error of triplicate dishes, and where not seen, error was too small for visual display

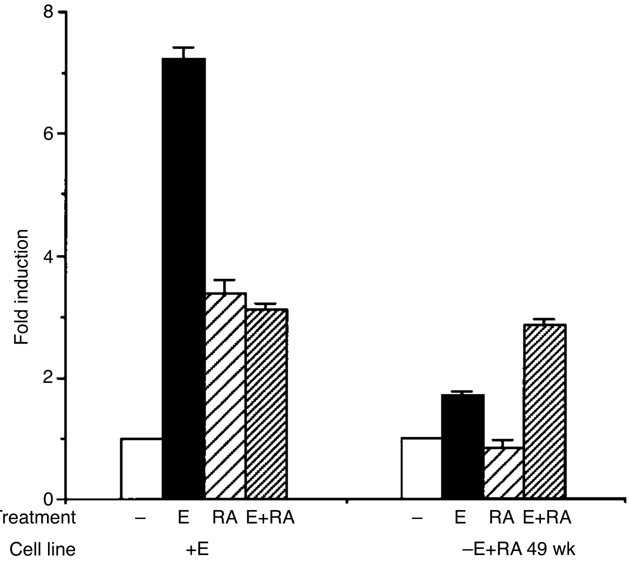

**Figure 6** Effect of long-term retinoic acid treatment on function of oestrogen receptors in ZR-75-1 human breast cancer cells. Function of receptors was assayed by transient transfection of an ERE-CAT gene. Cells were maintained either as stock cultures with $10^{-8}$ M oestradiol (cell line +E), or under conditions of steroid deprivation (phenol red-free RPMI 1640/5% DCFCS) with $10^{-6}$ M all-*trans* retinoic acid (cell line –E+RA) for the length of time indicated. Short-term expression of the transfected gene was then assessed as described in 'Materials and Methods' in phenol red-free RPMI 1640/5% DCFCS medium alone (–) (open bars), or supplemented with $10^{-8}$ M oestradiol (E) (solid bars), with $10^{-6}$ M all-*trans* retinoic acid (RA) (wide lines), with $10^{-8}$ M oestradiol and $10^{-6}$ M all-*trans* retinoic acid (E+RA) (close lines). CAT activity was expressed as pmol of $^{14}$C-acetyl group transferred from $^{14}$C-acetyl CoA to chloramphenicol per hour and normalized per unit of luciferase activity. All results are expressed as a fold induction ratio of treatment versus no treatment. Bars indicate the standard error of triplicate dishes of both no treatment and treatment acid treatment did result here in reduced ability for oestrogen to induce CAT activity from a transiently transfected ERE-CAT gene (Figure 6), as in the MCF7 cells (Figure 4).

### Expression of IGFBP3 after long-term RA exposure

Since retinoic acid inhibition of cell growth is thought to be associated with increased levels of growth inhibitory IGFBP3 (see introduction), levels of IGFBP3 were measured by Western ligand blotting in the long-term steroid deprived and retinoic acid treated cells. Stock oestrogen-maintained cells produced low levels of several IGFBP. The largest band, IGFBP3, was strongly increased by $10^{-6}$ M all-*trans* retinoic acid not only in both the MCF7 and ZR-75-1 oestrogen-maintained stock cells but also in the long-term oestrogen deprived MCF7 cells (Figure 7, tracks 1–8, 13–16). Long-term retinoic acid treated ZR-75-1 cells had only low levels of IGFBP3 equivalent to those seen without retinoic acid in the stock cells, whereas long-term retinoic acid treated MCF7 cells showed higher levels of IGFBP3 which were not reduced by short-term removal of retinoic acid or oestradiol administration (Figure 7, tracks 9–12, 17–20).

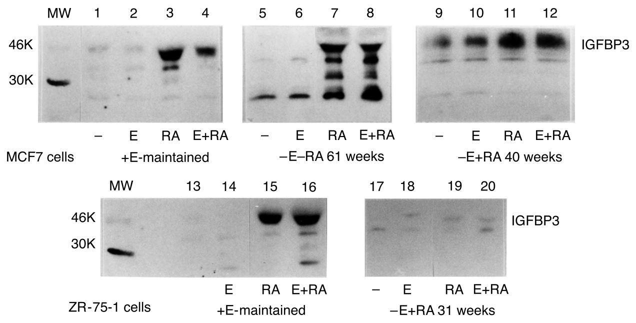

**Figure 7** Regulation of IGFBP in MCF7 and ZR-75-1 human breast cancer cells following long-term retinoic acid treatment. Western $^{125}$I-IGF1 ligand blot analysis of oestrogen-maintained cells (tracks 1–4 and 13–16), or of cells following steroid deprivation (phenol red-free RPMI 1640/5% DCFCS medium) (tracks 5–8), or of cells following steroid deprivation in the presence of $10^{-6}$ M all-*trans* retinoic acid (tracks 9–12 and 17–20). Short-term regulation of IGFBP was assessed in each stock line following growth for 7 days in RPMI 1640/5% DCFCS medium alone (–) (tracks 1, 5, 9, 13, 17), or supplemented with $10^{-8}$ M oestradiol (E) (tracks 2, 6, 10, 14, 18), with $10^{-6}$ M all-*trans* retinoic acid (RA) (tracks 3, 7, 11, 15, 19), or with $10^{-8}$ M oestradiol and $10^{-6}$ M all-*trans* retinoic acid (E+RA) (tracks 4, 8, 12, 16, 20). The positions of $^{14}$C-labelled protein molecular weight markers (MW) are shown in the left hand lanes

**Table 1** Effect of $10^{-6}$ M all-*trans* retinoic acid (RA) on induction of CAT activity from a transiently transfected AP1-CAT gene in MCF7 and ZR-75-1 human breast cancer cells in monolayer culture

| Long-term treatment of cells | With or without RA in CAT assay | CAT activity (pmol $^{14}$C-acetyl/unit luciferase/h) |
|---|---|---|
| MCF7 | –RA | 6.8 ± 0.6 |
| (E-maintained) | +RA | 3.6 ± 0.6 |
| MCF7 | –RA | 18.7 ± 0.8 |
| (+RA 66 wks) | +RA | 29.8 ± 4.0 |
| ZR-75-1 | –RA | 101.5 ± 3.3 |
| (E-maintained) | +RA | 63.9 ± 5.8 |
| ZR-75-1 | –RA | 266.8 ± 15.7 |
| (+RA 48 wks) | +RA | 322.4 ± 12.2 |

### AP1 activity after long-term RA exposure

Level of AP1 activity is thought to play a role in retinoic acid sensitivity in breast cancer cells (van der Burg et al, 1995). Transient transfection of an AP1-CAT inducible reporter gene into stock oestrogen-maintained MCF7 and ZR-75-1 cells showed that AP1 activity was decreased in both cases by the administration of $10^{-6}$ M all-*trans* retinoic acid (Table 1), although it was evident that the basal level of AP1 activity was much greater in the

 © 2000 Cancer Research Campaign

ZR-75-1 cells. Long-term retinoic acid treatment resulted in an altered AP1 response to retinoic acid in both cell lines, such that retinoic acid became stimulatory rather than inhibitory (Table 1).

### DISCUSSION

This paper has described the long-term effects of all-*trans* retinoic acid on two oestrogen-dependent human breast cancer cell lines MCF7 and ZR-75-1. Although both cell lines were inhibited by retinoic acid in the short term in either the absence or the presence of oestradiol, prolonged culture with retinoic acid resulted in the cells acquiring resistance to the growth inhibitory effects of retinoic acid. Long term culture in the presence of 1 μM all-*trans* retinoic acid resulted in reproducible upregulation of growth as time increased, until the cells were no longer growth inhibited by retinoic acid in either the absence or in the presence of oestradiol. Loss of response to retinoic acid was also accompanied by a loss of response to the growth stimulatory effects of oestradiol, although the time courses were separable. The time courses of loss of growth response to oestrogen under conditions of steroid deprivation have been documented previously for T47D and ZR-75-1 cells (Daly and Darbre, 1990). Steroid deprivation of MCF7 cells showed here a similar pattern of upregulation of basal non-oestrogen stimulated growth rate such that the cells learned to grow at the same rate in the absence of oestrogen as they grew with oestradiol throughout. However, loss of oestrogen growth response took less time in the MCF7 cells (15 weeks) than with T47D cells (32 weeks) (Daly and Darbre, 1990) or with ZR-75-1 cells (35–40 weeks) (Daly and Darbre, 1990). Addition of retinoic acid to the steroid deprived conditions preserved the oestrogen growth response of the MCF7 cells from 15 weeks to greater than 36 weeks (36–55 weeks), and was therefore separable from the loss of growth inhibitory effects of all-trans retinoic acid which had occurred already at 36 weeks.

Study of the outgrowth of cells under conditions of steroid deprivation and retinoic acid treatment in unsubcultured dishes revealed that the progression to resistance was clonal but it was reproducible in that many clones were visible on every dish with increasing time, and both individual clones and mixed unsubcultured cell populations followed similar time courses. This reinforces previous suggestions (Daly and Darbre, 1990) that loss of response results from adaptive growth changes which occur within a specific time frame for each cell line. Unfortunately the time frame was not the same for different cell lines, presumably due to the prevailing cellular context and in line with clinical observations of different lengths of remission to endocrine treatment in different patients (Miller, 1996).

The generation of cell populations all derived from the same parental cell line at various time points on the pathway to oestrogen resistance and retinoic acid resistance provides a cell biological model system to investigate molecular mechanisms. Loss of response to either oestrogen or retinoic acid did not result from loss of oestrogen receptors as measured by steroid binding assay. In fact, ER levels were increased in MCF7 cells 10-fold following long-term steroid deprivation and 6-fold in the steroid deprived retinoic acid-treated cells. This is in line with the ability of both oestrogen (Saceda et al, 1988) and retinoic acid (Rubin et al, 1994) to downregulate ER levels. Nor was loss of response to retinoic acid associated with loss of RARα mRNA as measured by Northern blotting, despite the fact that this receptor is thought to be a key mediator of retinoic acid growth inhibitory effects in MCF7 cells (Dawson et al, 1995). Regulation of RARα mRNA was not obvious in the short term by either oestrogen or retinoic acid in the oestrogen-maintained cells, but when in the long-term oestrogen deprived cells the levels fell to undetectable, there was an obvious increase not only by short-term readddiction of oestrogen as reported previously (Roman et al, 1993) but also by retinoic acid itself. Although the short-term regulation of RARα mRNA in the steroid deprived cells was not as strong with retinoic acid as with oestrogen, long-term treatment with retinoic acid could clearly compensate for lack of oestrogen in maintaining RARα mRNA levels and these could not be obviously altered by short-term manipulations. Thus, although retinoic acid resistance in ER negative breast cancer cell lines has been linked to underexpression of RARα mRNA (van der Burg, 1993), progression to retinoic acid resistance could occur here by adaptation within the same cell line without loss of RARα mRNA. Other studies (data not shown here but published in a PhD thesis (Stephen, 1998)) showed also by Northern blotting that neither RARγ nor RXRα mRNA levels decreased in the long-term retinoic acid treated cells compared with the stock oestrogen-maintained MCF7 cells. Published work has shown that overexpression of RARβ can restore retinoic acid sensitivity to the ER negative breast cancer cell line MDA-MB-231 (Li et al, 1995) and it would be interesting to explore any role for this receptor in the context of the ER positive MCF7 cells which have developed retinoic acid resistance.

Despite the continued presence of receptors ER/RARα, their function was reduced in some assays. As reported previously for steroid deprived ZR-75-1 cells (Daly and Darbre, 1990), Northern blotting of oestrogen-regulated pS2 mRNA showed no loss of function of ER in either steroid deprived or retinoic acid acid treated MCF7 cells. Furthermore, pS2 was increased not only by oestradiol but also by retinoic acid in the long-term steroid deprived cells. Inhibitory effects of retinoic acid on oestrogen action at the ERE level have been reported previously (Demirpence et al, 1994), but here we have noted positive effects of retinoic acid on an oestrogen-regulated gene in the absence of oestrogen. In contrast to endogenous pS2 gene expression, levels of induction of transiently transfected ERE-CAT and RRE-LUC reporter genes were reduced in the steroid deprived and retinoic acid treated cells but the reasons remain unknown.

Our results confirm previous work showing that retinoic acid treatment increases secretion of IGFBP3 into the medium conditioned by stock oestrogen maintained retinoic-inhibited MCF7 or ZR-75-1 cells. This was also true for the steroid deprived MCF7 cells and occurred in both the absence and the presence of oestradiol. However, the progression to retinoic acid growth resistance occurred in the long-term retinoic acid treated cells irrespective of continued high levels of IGFBP3 secreted from the MCF7 cells or reduced levels of IGFBP3 from the ZR-75-1 cells. If IGFBP3 is a mediator of retinoic acid-induced growth inhibition in breast cancer cells as some studies strongly indicate (Adamo et al, 1992; Oh, 1998; Shang et al, 1999), then our data would suggest that progression to resistance must be able to occur through some independent overriding pathway.

Transcriptional activity of AP1-linked genes has been another suggested site for interaction of retinoic acid and growth factor regulatory pathways (Gottlicher et al, 1998). Activity of the transcription factor AP1 is induced by mitogenic growth factors (Lamph et al, 1988) while growth inhibitors such as retinoids decrease its activity (Chen et al, 1995) through specific protein–protein interactions (DiSepio et al, 1999). In ER positive retinoic

© 2000 Cancer Research Campaign acid sensitive breast cancer cells, retinoic acid can inhibit AP1 activity while in ER negative retinoic acid resistant breast cancer cells retinoic acid could not alter AP1 activity (van der Burg et al, 1995). Furthermore, the antiproliferative effects of retinoic acid can be overcome in MCF7 cells by overexpression of c-jun (Yang et al, 1997). Our results here confirm that AP1 activity is reduced by retinoic acid in oestrogen-maintained retinoic acid sensitive breast cancer cells, but not in their long-term retinoic acid maintained and resistant counterparts. Comparison of MCF7 and ZR-75-1 cell lines showed that different ER positive cell lines have markedly different basal AP1 activities and thus that level of AP1 activity did not equate with growth resistance to retinoic acid. However, comparison of the retinoic acid sensitive with resistant counterparts of the same parental origin does suggest a correlation between loss of growth response to retinoic acid and loss of the ability of retinoic acid to reduce endogenous AP1 activity. Progression to retinoic acid resistance may thus be accompanied by a lost ability of retinoic acid to reduce AP1 levels rather than relate to any absolute value for AP1 levels. The mechanisms remain unknown, but could relate to altered metabolism rendering the retinoic acid ineffective (Takatsuka et al, 1996), and are in line with other recent data linking the multidrug resistant (MDR) phenotype with increased AP1 activity (Daschner et al, 1999).

If there is any validity in extrapolation from cell culture to human tumours, then these results warn of potential resistance which could arise on long term treatment with retinoic acid in the clinical situation, and echo the problems of progression to endocrine resistance (Miller, 1996). However, since resistance to retinoic acid was separable from resistance to oestrogen, and since retinoic acid could delay progression to oestrogen resistance, this may offer the opportunity for development of alternating alternative therapies. Furthermore, since progression to retinoic acid resistance was concentration-dependent, progression can no longer be considered as absolute but rather as a mechanism of escape from prevailing environmental conditions. Oestrogen resistance has recently been shown also to follow a similar course to altered rather than lost sensitivity (Jeng et al, 1998). This may offer further clinical possibilities for manipulation of dose as well as type of therapy.

### ACKNOWLEDGEMENTS

This research was supported financially by the Felix Foundation and ORS award (RS). We thank Mrs E Hales for technical assistance. We thank Professor R Evans (The Salk Institute, California, USA) for the RAR and RXR cDNAs and Professor P Chambon (Centre National de la Recherche Scientifique, Strasbourg, France) for the pS2 and 36B4 cDNAs. We thank Dr M Parker (Imperial Cancer Research Fund, London) for the ERE-CAT and RRE-LUC vectors and Dr DR Soprano (Temple University, Philadelphia, USA) for the AP1-CAT vector.

### REFERENCES

Adamo ML, Shao ZM, Lanau F, Chen JC, Clemmons DR, Roberts CT, LeRoith D and Fontana JA (1992) Insulin-like growth factor-1 (IGF1) and retinoic acid modulation of IGF-binding proteins (IGFBPs): IGFBP-2, -3, and -4 gene expression and protein secretion in a breast cancer cell line. *Endocrinol* **131**: 1858–1866

Anzano MA, Byers SW, Smith JM, Peer CW, Mullen LT, Brown CC, Roberts AB and Sporn MB (1994) Prevention of breast cancer in the rat with 9-*cis*-retinoic acid as a single agent and in combination with tamoxifen. *Cancer Res* **54**: 4614–4617

Brown AMC, Jeltsch JM, Roberts M and Chambon P (1984) Activation of pS2 gene transcription is a primary response to estrogen in the human breast cancer cell line MCF-7. *Proc Natl Acad Sci USA* **81**: 6344–6348

Budd GT, Adamson PC, Gupta M, Homayoun P, Sandstrom SK, Murphy RF, McLain D, Tuason L, Peereboom D, Bukowski RM and Ganapathi R (1998) Phase I/II trial of all-trans retinoic acid and tamoxifen in patients with advanced breast cancer. *Clin Cancer Res* **4**: 635–642

Butler WB and Fontana JA (1992) Responses to retinoic acid of tamoxifen-sensitive and -resistant sublines of human breast cancer cell line MCF-7. *Cancer Res* **52**: 6164–6167

Chambon P (1996) A decade of molecular biology of retinoic acid receptors. *FASEB J* **10**: 940–954

Chen JY, Penco S, Ostrowski J, Balaguer P, Pons M, Starrett JE, Reczek P, Chambon P and Gronemeyer H (1995) RAR-specific agonist/antagonists which dissociate transactivation and AP1 transrepression inhibit anchoarge-independent cell proliferation. *EMBO J* **14**: 1187–1197

Costa A (1993) Breast cancer chemoprevention. *Eur J Cancer* **29A**: 589–592

Daly RJ and Darbre PD (1990) Cellular and molecular events in loss of estrogen sensitivity in ZR-75–1 and T-47-D human breast cancer cells. *Cancer Res* **50**: 5868–5875

Daly RJ, King RJB and Darbre PD (1990) Interaction of growth factors during progression towards steroid independence in T-47-D human breast cancer cells. *J Cell Biochem* **43**: 199–211

Darbre PD and Daly RJ (1989) Effects of oestrogen on human breast cancer cells in culture. *Proc Roy Soc Edin* **95B**: 119–132

Darbre P, Yates J, Curtis S and King RJB (1983) Effect of estradiol on human breast cancer cells in culture. *Cancer Res* **43**: 349–354

Daschner PJ, Ciolino HP, Plouzek CA and Yeh GC (1999) Increased AP-1 activity in drug resistant human breast cancer MCF-7 cells. *Breast Cancer Res Treat* **53**: 229–240

Dawson MI, Chao WR, Pine P, Jong L, Hobbs PD, Rudd CK, Quick TC, Niles RM, Zhang XK, Lombardo A, Ely KR, Shroot B and Fontana JA (1995) Correlation of retinoid binding affinity to retinoic acid receptor α with retinoid inhibition of growth of estrogen receptor-positive MCF-7 mammary carcinoma cells. *Cancer Res* **55**: 4446–4451

Demirpence E, Balaguer P, Trousse F, Nicolas JC, Pons M and Gagne D (1994) Antiestrogenic effects of all-*trans* retinoic acid and 1,25-dihydroxyvitamin $D_3$ in breast cancer cells occur at the estrogen response element level but through different molecular mechanisms. *Cancer Res* **54**: 1458–1464

DiSepio D, Sutter M, Johnson AT, Chandraratna RA and Nagpal S (1999) Identification of the AP1-antagonism domain of retinoic acid receptors. *Mol Cell Biol Res Commun* **1**: 7–13

Engel LW, Young NA, Trolka TS, Lippman ME, O'Brian SJ and Joyce MJ (1978) Establishment and characterisation of three new continuous cell lines derived from human breast carcinomas. *Cancer Res* **38**: 3352–3364

Fontana JA (1987) Interaction of retinoids and tamoxifen on the inhibition of human mammary carcinoma cell proliferation. *Exp Cell Biol* **55**: 136–144

Fontana JA, Mezu AB, Cooper BN and Miranda D (1990) Retinoid modulation of estradiol-stimulated growth and of protein synthesis and secretion in human breast carcinoma cells. *Cancer Res* **50**: 1997–2002

Fontana JA, Burrows-Mezu A, Clemmons DR and LeRoith D (1991) Retinoic modulation of insulin-like growth binding proteins and inhibition of breast carcinoma proliferation. *Endocrinol* **128**: 1115–1122

Fontana JA, Nervi C, Shao ZM and Jetten AM (1992) Retinoid antagonism of estrogen-responsive transforming growth factor alpha and pS2 gene expression in breast carcinoma cells. *Cancer Res* **52**: 3938–3945

Gottlicher M, Heck S and Herrlich P (1998) Transcriptional cross-talk, the second mode of steroid hormone receptor action. *J Mol Med* **76**: 480–489

Green B and Leake RE (editors) (1987) *Steroid Hormones: A Practical Approach*. IRL Press, Oxford, UK

Grubbs CJ, Moon RC, Sporn MB and Newton DL (1977) Inhibition of mammary cancer by retinyl methyl ester. *Cancer Res* **37**: 599–602

Hossenlopp P, Seurin D, Quinson BS, Hardouin S and Binoux M (1986) Analysis of serum insulin-like growth factor binding proteins using Western blotting: use of the method for titration of the binding proteins and competitive binding studies. *Anal Biochem* **154**: 138–143

Jeng MH, Shupnik MA, Bender TP, Westin EH, Bandyopadhyay D, Kumar R, Masamura S and Santen RJ (1998) Estrogen receptor expression and function in long-term estrogen-deprived human breast cancer cells. *Endocrinol* **139**: 4164–4174

Katzenellenbogen BS, Kendra KL, Norman MJ and Berthois Y (1987) Proliferation, hormonal responsiveness, and estrogen receptor content of MCF-7 human

*© 2000 Cancer Research Campaign*

breast cancer cells grown in the short-term and long-term absence of estrogens. *Cancer Res* **47**: 4355–4360

Kazmi SMI, Plante RK, Visconti V and Lau CY (1996) Comparison of N-(4-hydroxyphenyl)retinamide and all-*trans* retinoic acid in the regulation of retinoid receptor-mediated gene expression in human breast cancer cell lines. *Cancer Res* **56**: 1056–1062

Kizaki M, Fukuchi Y and Ikeda Y (1999) A novel retinoic acid-resistant acute promyelocytic leukemia model in vitro and in vivo (review). *Int J Mol Med* **4**: 359–364

Koga M and Sutherland RL (1991) Retinoic acid acts synergistically with 1,25-dihydroxyvitamin $D_3$ or antioestrogen to inhibit T47D human breast cancer cell proliferation. *J Steroid Biochem Molec Biol* **39**: 455–460

Lacroix A and Lippman ME (1980) Binding of retinoids to human breast cancer cell lines and their effects on cell growth. *J Clin Invest* **65**: 586–591

Lacroix A, L'Heureux N and Bhat PV (1984) Cytoplasmic retinoic acid-binding protein in retinoic acid-resistant human breast cancer sublines. *J Natl Cancer Inst* **73**: 793–800

Lamph WW, Wamsley P, Sassone-Corsi P and Verma IM (1988) Induction of protooncogene Jun/AP1 by serum and TPA. *Nature* **334**: 629–631

Larssen SS, Madsen MW, Jensen BL and Lykesfeldt AE (1997) Resistance of human breast-cancer cells to the pure steroidal anti-oestrogen ICI 182,780 is not associated with a general loss of estrogen-receptor expression or lack of estrogen responsiveness. *Int J Cancer* **72**: 1129–1136

Li XS, Shao ZM, Sheikh MS, Eiseman JL, Sentz D, Jetten AM, Chen JC, Dawson MI, Aisner S, Rishi AK, Gutierrez P, Schnapper L and Fontana JA (1995) Retinoic acid nuclear receptor β inhibits breast carcinoma anchorage independent growth. *J Cell Physiol* **165**: 449–458

Lotan R (1979) Different susceptibilities of human melanoma and breast cancer cell lines to retinoic acid-induced growth inhibition. *Cancer Res* **39**: 1014–1019

Marth B, Bock G and Daxenbichler G (1985) Effect of 4-hydroxyphenylretinamide and retinoic acid on proliferation and cell cycle of cultured human breast cancer cells. *J Natl Cancer Inst* **75**: 871–875

Masiakowski P, Breathnach R, Bloch J, Gannon F, Krust A and Chambon P (1982) Cloning of cDNA sequences of hormone-regulated genes from the MCF-7 human breast cancer cell line. *Nucleic Acids Res*, **10**: 7895–7903

Miller WR (1996) *Estrogen and Breast Cancer*. Chapman and Hall

Moon RC, Grubbs CJ and Sporn MB (1976) Inhibition of 7,12-dimethyl benz (α) anthracene-induced mammary carcinogenesis by retinyl acetate. *Cancer Res* **36**: 2627–2630

Moon RC, Grubbs CJ, Sporn MB and Goodman DG (1977) Retinyl-acetate inhibits mammary carcinogenesis induced by N-methyl-N-nitrosourea. *Nature* **267**: 620–621

Oh Y (1998) IGF-independent regulation of breast cancer growth by IGF binding proteins. *Breast Cancer Res Treat* **47**: 283–293

Osborne CK, Hobbs K and Trent JM (1987) Biological differences among MCF-7 human breast cancer cell lines from different laboratories. *Breast Cancer Res Treat* **9**: 111–121

Rettura G, Schittek A, Hardy M, Levenson SM, Demetriou A and Seifter E (1975) Antitumour action of vitamin A in mice inoculated with adenocarcinoma cells. *J Natl Cancer Inst* **54**: 1489–1491

Rishi AK, Shao ZM, Baumann RG, Li XS, Sheikh MS, Kimura S, Bashirelahi N and Fontana JA (1995) Estradiol regulation of the human retinoic acid receptor α gene in human breast carcinoma cells is mediated via an imperfect half-palindromic estrogen response element and Sp1 motifs. *Cancer Res* **55**: 4999–5006

Roman SD, Clarke CL, Hall RE, Alexander IE and Sutherland RL (1992) Expression and regulation of retinoic acid receptors in human breast cancer cells. *Cancer Res* **52**: 2236–2242

Roman SD, Ormandy CJ, Manning DL, Blamey RW, Nicholson RI, Sutherland RL and Clarke CL (1993) Estradiol induction of retinoic acid receptors in human breast cancer cells. *Cancer Res* **53**: 5940–5945

Rubin M, Fenig E, Rosenauer A, Menendez-Botet C, Achkar C, Bentel JM, Yahalom J, Mendelsohn J and Miller WH (1994) 9-*cis* retinoic acid inhibits growth of breast cancer cells and down-regulates estrogen receptor RNA and protein. *Cancer Res* **54**: 6549–6556

Saceda M, Lippman ME, Chambon P, Lindsey RL, Ponglikitmongkol M, Puente M and Martin MB (1988) Regulation of the estrogen receptor in MCF-7 cells by estradiol. *Mol Endocrinol* **2**: 1157–1162

Salacinski PRP, McLean C, Sykes JEC, Clement-Jones VV and Lowry PJ (1981) Iodination of proteins, glycoproteins, and peptides using a solid phase oxidising agent, 1,3,4,6-tetrachloro-3,6-diphenyl glycouril (Iodogen). *Anal Biochem* **117**: 136–146

Sambrook J, Fritsch EF and Maniatis T (1989) *Molecular Cloning: A Laboratory Manual*. Cold Spring Harbor Laboratory Press, New York, USA

Shang Y, Baumrucker CR and Green MH (1999) Signal relay by retinoic acid receptors α and β in the retinoic acid-induced expression of insulin-like growth factor-binding protein-3 in breast cancer cells. *J Biol Chem* **274**: 18005–18010

Sheikh MS, Shao ZM, Chen JC, Hussain A, Jetten AM and Fontana JA (1993) Estrogen receptor-negative breast cancer cells transfected with the estrogen receptor exhibit increased RAR alpha gene expression and sensitivity to growth inhibition by retinoic acid. *J Cell Biochem* **53**: 394–404

Sheikh MS, Shao ZM, Li XS, Dawson M, Jetten AM, Wu S, Conley BA, Garcia M, Rochefort H and Fontana JA (1994) Retinoid-resistant estrogen receptor-negative human breast carcinoma cells transfected with retinoic acid receptor-alpha acquire sensitivity to growth inhibition by retinoids. *J Biol Chem* **269**: 21440–21447

Slack JL (1999) Biology and treatment of acute progranulocytic leukemia. *Curr Opin Hematol* **6**: 236–240

Sleigh MJ (1986) A nonchromatographic assay for expression of the chloramphenicol acetyltransferase gene in eucaryotic cells. *Anal Biochem* **156**: 251–256

Soprano DR, Chen LX, Wu S, Donigan AM, Borghaei RC and Soprano KJ (1996) Overexpression of both RAR and RXR restores AP-1 repression in ovarian adenocarcinoma cells resistant to retinoic acid-dependent growth inhibition. *Oncogene* **12**: 577–584

Stephen RL (1998) *The role of retinoic acid in the growth regulation of human breast cancer cells*. Ph D Thesis, The University of Reading, UK

Takatsuka J, Takahashi N and De Luca LM (1996) Retinoic acid metabolism and inhibition of cell proliferation: an unexpected liaison. *Cancer Res* **56**: 675–678

Ueda H, Ono M, Hagino Y and Kuwano M (1985) Isolation of retinoic acid-resistant clones from human breast cancer cell line MCF-7 with altered activity of cellular retinoic acid-binding protein. *Cancer Res* **45**: 3332–3338

Umesono K, Murakami KK, Thompson CC and Evans RM (1991) Direct repeats as selective response elements for the thyroid hormone, retinoic acid, and vitamin D3 receptors. *Cell* **65**: 1255–1266

van der Burg B, van der Leede BM, Kwakkenbos-Isbrucker L, Salverda S, de Laat SW, van der Saag PT (1993) Retinoic acid resistance of estradiol-independent breast cancer cells coincides with diminished retinoic acid receptor function. *Mol Cell Endocrinol* **91**: 149–157

van der Burg B, Slager-Davidov R, van der Leede BM, de Laat SW and van der Saag PT (1995) Differential regulation of AP1 activity by retinoic acid in hormone-dependent and -independent breast cancer cells. *Mol Cell Endocrinol* **112**: 143–152

Wakeling AE (1993) Are breast tumours reistant to tamoxifen also resistant to pure antioestrogens? *J Steroid Biochem Molec Biol* **47**: 107–114

Welshons WV and Jordan VC (1987) Adaptation of estrogen-dependent MCF-7 cells to low estrogen (phenol red-free) culture. *Eur J Cancer Clin Oncol* **23**: 1935–1939

Wetherall NT and Taylor CM (1986) The effects of retinoid treatment and antiestrogens on the growth of T47D human breast cancer cells. *Eur J Cancer Clin Oncol* **22**: 53–59

Wigler M, Pellicer A, Silverstein S, Axel R, Urlaub G and Chasin L (1979) DNA mediated transfer of the adenine phosphoribosyltransferase locus into mammalian cells. *Proc Natl Acad Sci USA* **76**: 1373–1376

Yang LM, Kim HT, Munoz-Medellin D, Reddy P and Brown PH (1997) Induction of retinoid resistance in breast cancer cells by overexpression of c-jun. *Cancer Res* **57**: 4652–4661

*© 2000 Cancer Research Campaign*
